# Shared diagnostic genes and potential mechanisms between intervertebral disc degeneration and diabetes mellitus revealed by integrated transcriptomic analysis and machine learning

**DOI:** 10.3389/fendo.2025.1576826

**Published:** 2025-07-18

**Authors:** Bao Song, Jianmin Wang, Hao Tang, Huadong Li, Wanli Zhang

**Affiliations:** Department of Tuina, Affiliated Hospital of Shandong University of Traditional Chinese Medicine, Jinan, China

**Keywords:** intervertebral disc degeneration, diabetes mellitus, PRTN3, diagnostic, immune infiltration

## Abstract

**Introduction:**

Intervertebral disc degeneration (IDD) and diabetes mellitus (DM) are clinically associated beyond traditional risk factors, yet the shared molecular mechanisms remain unclear.

**Methods:**

We integrated transcriptomic data from IDD and DM cohorts, performed differential expression and protein–protein interaction (PPI) network analyses, and applied machine learning to identify shared diagnostic genes. Pathway enrichment, immune infiltration analyses, and experimental qPCR validation were conducted to explore mechanisms and confirm findings.

**Results:**

We identified 138 shared differentially expressed genes enriched in immune-related pathways. Seven hub genes, including PRTN3, were identified by Random Forest models. PRTN3 showed consistent upregulation across discovery, validation, and internal cohorts. Pathway and immune analyses revealed strong associations between PRTN3 expression and neutrophil-related processes in both IDD and DM. Experimental validation confirmed PRTN3 upregulation in blood samples from patients with concurrent IDD and DM.

**Discussion:**

Our findings suggest that immune-inflammatory mechanisms, particularly involving neutrophils, underlie the comorbidity between IDD and DM. PRTN3 emerges as a promising shared diagnostic biomarker and potential therapeutic target for these conditions.

## 1 Introduction

Intervertebral disc degeneration (IDD) and diabetes mellitus (DM) are two common chronic diseases that severely impact patients’ quality of life and place a substantial burden on global healthcare systems. IDD is a major cause of low back pain (1), with a complex pathogenesis involving chronic mechanical overload, persistent inflammation, dysregulated matrix metalloproteinase (MMP) activity, and impaired disc nutrient metabolism (2–4). Moreover, growing evidence suggests a potential link between metabolic dysfunction and intervertebral disc degeneration (3). DM, characterized by chronic hyperglycemia, is a metabolic disease caused by impaired insulin secretion or function and is associated with a range of complications affecting the cardiovascular system, kidneys, retina, and nervous system (5, 6). Previous studies have demonstrated that patients with type 2 diabetes mellitus (T2DM) face an increased risk of musculoskeletal disorders, including osteoporosis, ligament laxity, and joint disease (7).

Epidemiological studies have revealed a correlation between DM and IDD, with a higher prevalence of disc disease in patients with DM (8), suggesting potential shared pathological mechanisms. Among these, chronic inflammation is considered a critical common mechanism. Both IDD and DM are inflammatory diseases in which chronic low-grade inflammation drives disc degeneration by promoting the release of pro-inflammatory cytokines and chemokines, leading to increased matrix metalloproteinase activity, which ultimately compromises disc structural integrity (9). Additionally, chronic inflammation contributes to pancreatic β-cell dysfunction and apoptosis, further exacerbating diabetes progression (10, 11). These findings highlight the essential role of immune-inflammatory interactions in the comorbid mechanisms of IDD and DM.

However, despite their evident association, the molecular mechanisms underlying their co-occurrence remain unclear, and no specific diagnostic biomarkers have been identified. Therefore, a combined analysis of lumbar disc herniation and diabetes not only provide insight into their potential connection but may also lead to new strategies for personalized diagnosis and treatment. By integrating bioinformatics-based analysis, it is possible to identify shared key genes and molecular mechanisms between the two diseases, as well as explore potential biomarkers for prognostic early diagnosis and prognosis evaluation. Furthermore, these findings may serve as a theoretical foundation for the development of joint therapeutic strategies, ultimately improving treatment efficacy and patients’ quality of life.

In summary, this study aimed to systematically analyze high-throughput transcriptomic data from patients with IDD and DM using integrative bioinformatics approaches. The goal was to explore potential shared molecular mechanisms and biological pathways, identify key genes and critical signaling pathways, and offer novel insights into the pathogenesis and potential treatment strategies for both diseases.

## 2 Methods

### 2.1 Data acquisition and preprocessing

**Figure 1:**
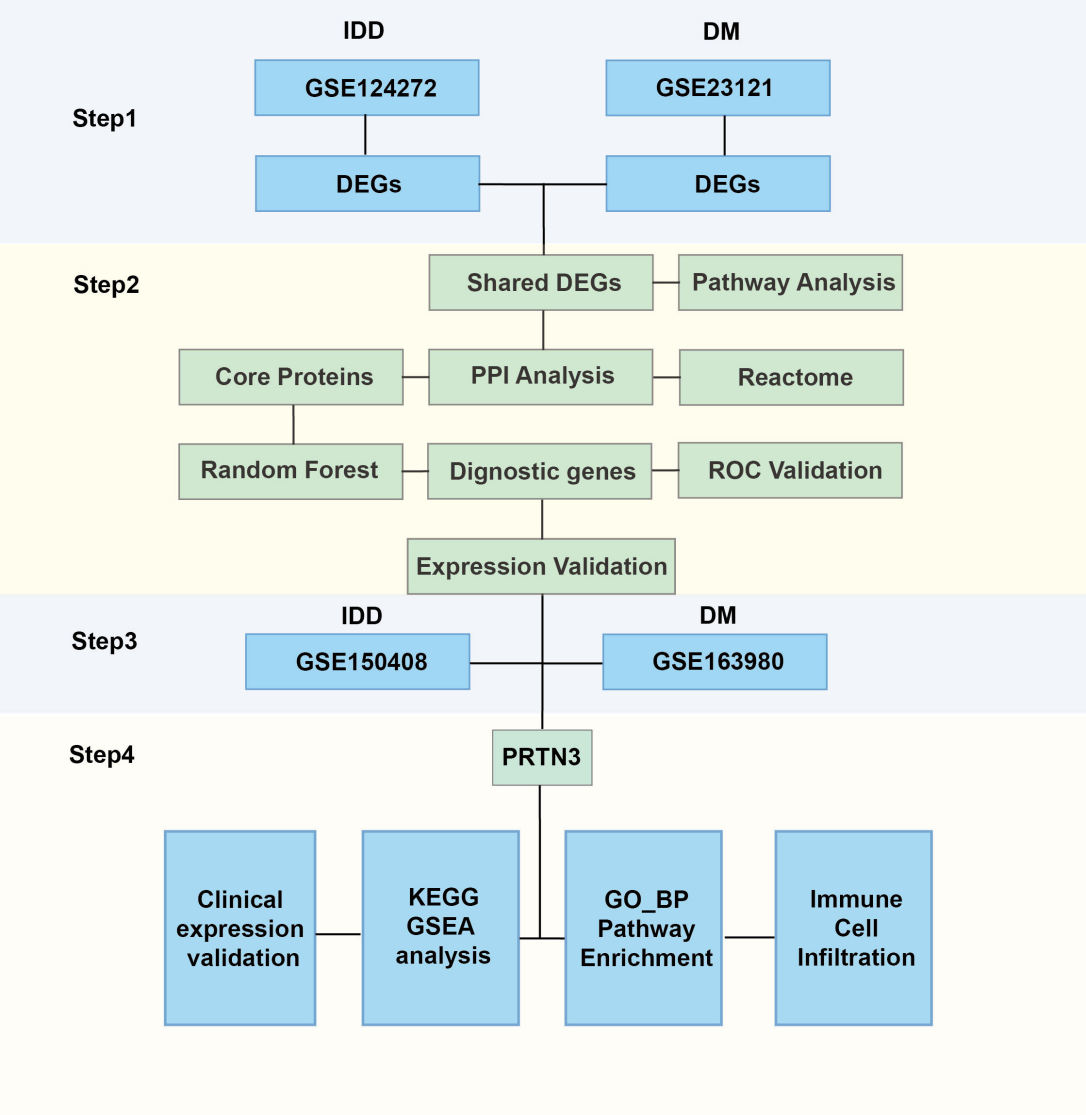
Research framework of this study.

The gene expression matrix and corresponding clinical data for intervertebral disc degeneration (IDD) and diabetes mellitus (DM) were obtained from the Gene Expression Omnibus (GEO, https://www.ncbi.nlm.nih.gov/geo/). For IDD, two microarray datasets were included: GSE124272, which contains 8 samples from patients with IDD and 8 healthy controls, and GSE150408, which includes 42 IDD samples and 17 control samples. The GSE124272 dataset was used as the discovery cohort, while GSE150408 served as the validation dataset for gene expression analysis. For DM, two additional microarray datasets were selected: GSE21321, consisting of 9 samples from patients with type 2 diabetes mellitus (T2DM) and 8 healthy controls, and GSE163980, comprising 5 T2DM samples and 5 control samples. Among these, GSE21321 was used as the discovery dataset, and GSE163980 was used for validation purposes. The “NormalizeBetweenArrays” function of the “limma” R package was used to eliminate batch effects (12). The overall research framework is depicted in **Figure 1**.

### 2.2 Differential expression genes analysis

To identify differentially expressed genes (DEGs) between the disease and control groups, we utilized the “limma” R package. DEGs were defined as genes with a p-value < 0.05 and a fold change threshold of |log2-FC| > 0.585. Volcano plots were generated by the “genekitr” R package to visualize the distribution of DEGs.

### 2.3 Pathway enrichment analysis

To explore the biological functions and signaling pathways associated with differentially expressed genes (DEGs), we conducted Gene Ontology (GO) and Kyoto Encyclopedia of Genes and Genomes (KEGG) pathway enrichment analyses using the “clusterProfiler” R package (13). Results with significantly enriched terms (P.adjust < 0.05) were visualized using the “genekitr” R package (14). Additionally, gene set enrichment analysis (GSEA) was performed to identify pathway enrichment in patients with varying levels of PRTN3 expression. KEGG pathways were utilized for GSEA, which was also conducted using the “genekitr” package. Enrichment results with P.adjust < 0.05 were considered statistically significant.

### 2.4 Protein-Protein Interaction Networks analysis

Proteins interact to form functional networks, and the more enriched these interactions are, the more central the biological functions tend to be. A total of 138 shared DEGs between IDD and DM were input into the STRING database (https://string-db.org) to construct a protein–protein interaction (PPI) network. Reactome pathway enrichment analysis was conducted using STRING (https://string-db.org) (15), with no specific parameters predefined. Based on the analysis, a total of 47 proteins with a degree score greater than the average were identified as core proteins and included in subsequent analyses.

### 2.5 Identification of hub biomarkers using machine learning

To identify disease-specific feature genes using machine learning, we employed the”randomForest” R package to classify important genes using the Random Forest (RF) algorithm. RF analysis, based on the decision tree approach, identified the most significant variables (genes). A random forest model with 500 trees was constructed for the corresponding cohort, and the optimal number of trees was determined by minimizing the cross-validation error. Genes were ranked by importance, and the top 20 most important genes were selected as feature genes. The intersection of disease-specific feature genes from both conditions was then identified.

### 2.6 Validation of the diagnostic performance of hub biomarkers

To evaluate the accuracy of the three hub biomarkers in the IDD and DM cohorts, the “pROC” R package was used to construct ROC curves, and the results were visualized using”ggplot2” (16). The IDD cohorts were obtained from GSE124272, while the validation cohort was sourced from GSE150408. For the DM group, the discovery cohort consisted of GSE21321, and the validation cohort was from GSE163980. The expression comparison of core diagnostic genes in DM and IDD was tested using a t-test and visualized using ggplot2.

### 2.7 Immune infiltration analysis

The “IOBR” R package was used to estimate immune cells in the DM and IDD cohorts (17). Specifically, the MPCounter algorithm was employed to quantify the relative immune cell fractions in individual samples. No specific parameters were manually set. A t-test was used for statistical analysis of immune cell differences between groups, and Pearson correlation was applied to calculate correlations.

### 2.8 Sample collection, mRNA extraction, and qPCR

Peripheral blood samples were collected from patients with DM and IDD comorbidities, as well as from healthy controls, using EDTA-coated tubes to prevent coagulation. All samples were processed within two hours of collection to ensure RNA integrity. Total RNA was extracted from blood samples using the MolPure^®^ Blood RNA Kit (Yeasen, China) according to the manufacturer’s instructions. The extracted RNA was used to synthesize the first strand cDNA using the Transcriptor High Fidelity cDNA Synthesis Kit (Roche, Switzerland). For PCR analysis, Green Taq Mix (TAKARA, China) was used according to the manufacturer’s instructions. The primers used in this study were as follows: PRTN3 Forward: 5’-CCTGCAGGAGCTCAATGT-3’,Reverse:5’-CTGAGTCTCCGAAGCAGATG-3’; GAPDH Forward: 5’-AGGGCTGCTTTTAACTCTGGT-3’, Reverse:5’-CCCCACTTGATTTTGGAGGGA-3’. For qRT-PCR analysis, the SYBR^®^ Green Premix Kit (TAKARA, China) was used according to the manufacturer’s instructions.

### 2.9 Statistical analysis

Data processing, analysis, and visualization were carried out using R packages (version 4.30; https://www.bioconductor.org/) or GraphPad Prism software (version 8.0.1; https://www.graphpad.com/). To compare differences between two groups, we employed Student’s t-test. Correlation analysis between variables was performed using Pearson’s correlation test. A significance level of P < 0.05 was considered statistically significant. Results are expressed as the mean ± standard error of the mean.

## 3 Results

### 3.1 Identification of DEGs in intervertebral disc degeneration and diabetes mellitus

**Figure 2:**
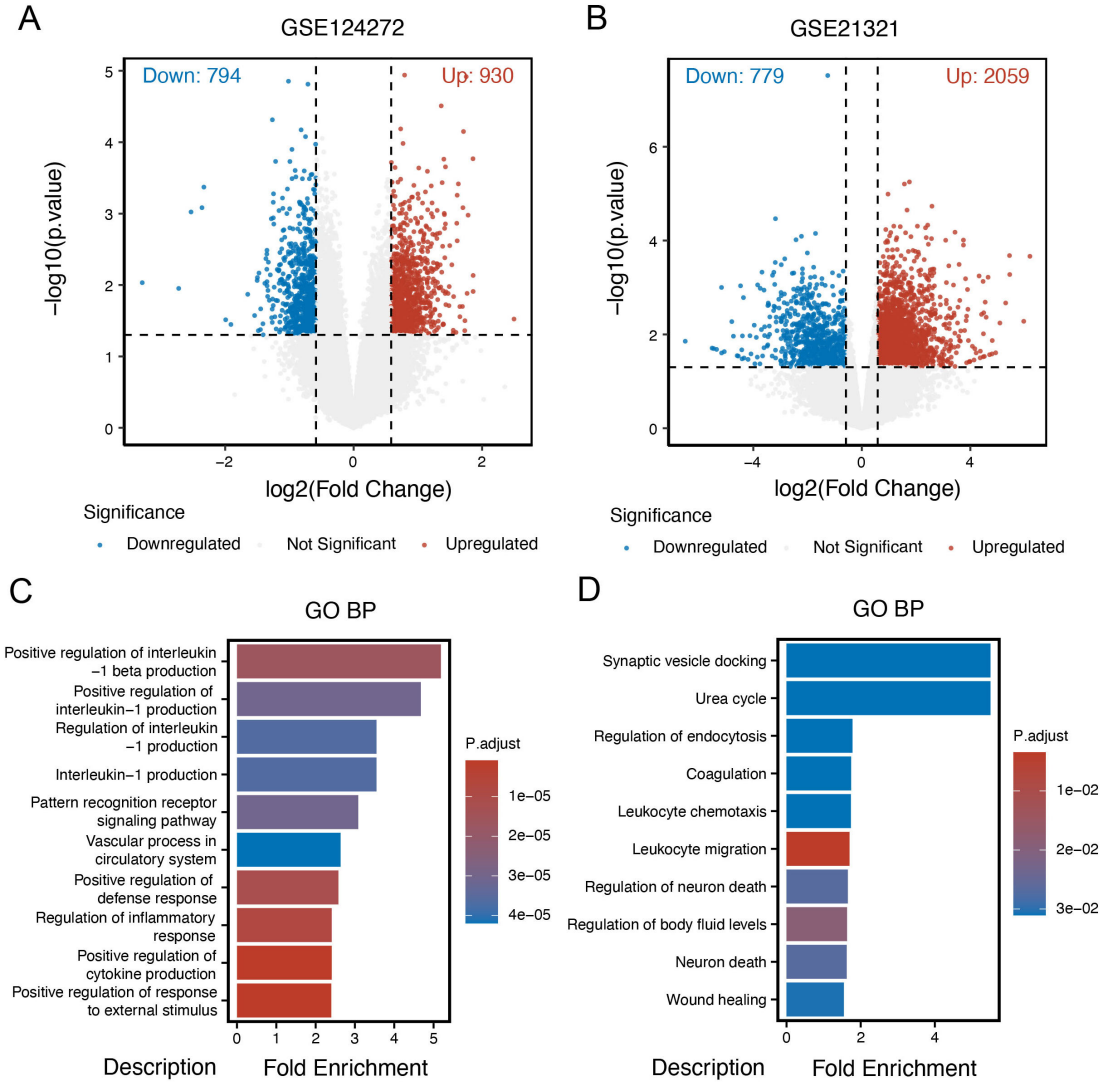
Identification of DEGs in intervertebral disc degeneration and diabetes mellitus. **(A)** Volcano plot of different expression genes (DEGs) in the intervertebral disc degeneration group compared to control cases. **(B)** Volcano plot of DEGs in the DM group compared to control cases. **(C)** Gene Ontology (GO) enrichment analysis of DEGs in the intervertebral disc degeneration group. **(D)** GO enrichment analysis of DEGs in the DM group.

Given the potential connection between intervertebral disc degeneration (IDD) and diabetes mellitus (DM), our study initially aimed to identify differentially expressed genes (DEGs) in both conditions. Using the “limma” R package, we characterized DEGs for each disease group. In the IDD group, a total of 1,724 DEGs were identified, including 930 upregulated and 794 downregulated genes. In the DM group, compared to control samples, 2,838 DEGs were identified, comprising 2,059 upregulated and 779 downregulated genes. Volcano plots were generated to visualize the distribution of DEGs in the IDD and DM cohorts (**Figures 2A, B**). Gene Ontology Biological Process (GOBP) enrichment analysis of DEGs in the IDD group revealed significant enrichment in immune-inflammatory pathways, such as the positive regulation of interleukin-1 beta production (**Figure 2C**). In contrast, enrichment analysis of DEGs in the DM group highlighted metabolic pathways, including the urea cycle process. Notably, immune-related pathways such as leukocyte chemotaxis and leukocyte migration were also significantly enriched in the DM group (**Figure 2D**), emphasizing that immune-related processes may serve as shared mechanisms underlying both IDD and DM.

### 3.2 Identification of core interacting proteins shared by intervertebral disc degeneration and diabetes mellitus

**Figure 3:**
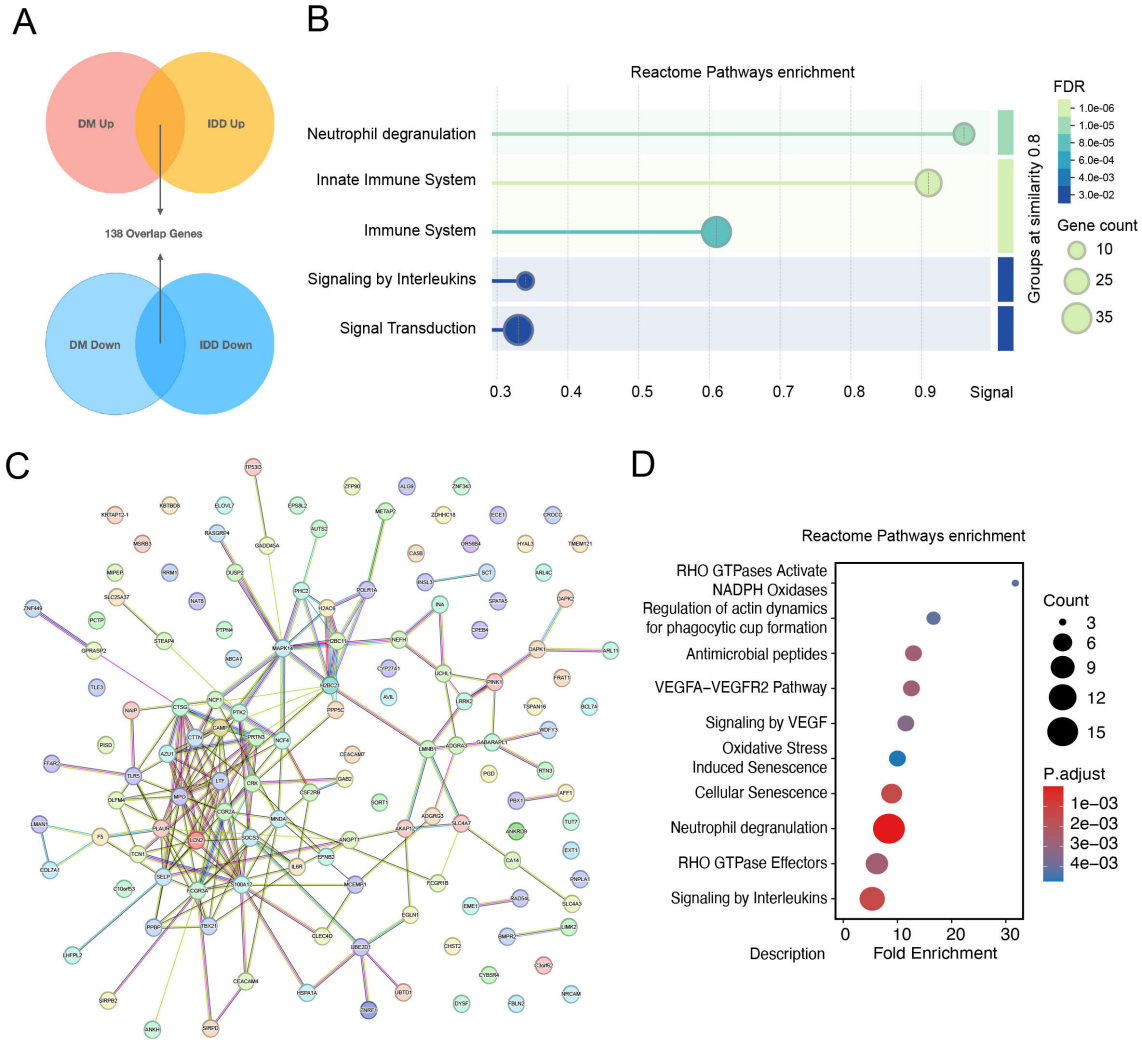
Identification of core interacting proteins shared by intervertebral disc degeneration and diabetes mellitus. **(A)** Determination of shared differentially expressed genes among intervertebral disc degeneration and diabetes. **(B)** Reactome pathways enrichment dotplot of 138 overlap genes. **(C)** Protein and protein interaction network of overlapping genes. **(D)** Dot Plot of Reactome pathway enrichment for core interaction proteins.

The shared genes between intervertebral disc degeneration (IDD) and diabetes mellitus (DM) are thought to contribute to the common pathogenic mechanisms of both conditions. A total of 138 differentially expressed genes (DEGs), either co-upregulated or co-downregulated in the IDD and DM groups, were identified (**Figure 3A**). Reactome pathway enrichment analysis of these shared DEGs revealed significant enrichment in immune-related processes, such as neutrophil degranulation, the immune system, and the innate immune system pathways. These findings underscore the critical role of immune-related processes in the shared pathogenesis of IDD and DM (**Figure 3B**). A protein–protein interaction (PPI) network was subsequently constructed to identify key interacting proteins among the shared DEGs (**Figure 3C**). A total of 47 core interacting proteins—defined by a degree value greater than or equal to the average—were identified. Reactome pathway analysis of these core proteins reaffirmed the significant enrichment of the neutrophil degranulation pathway (**Figure 3D**), suggesting a potential role for neutrophils in the pathophysiology of both IDD and DM.

### 3.3 Identify potential shared diagnostic genes based on machine learning

**Figure 4:**
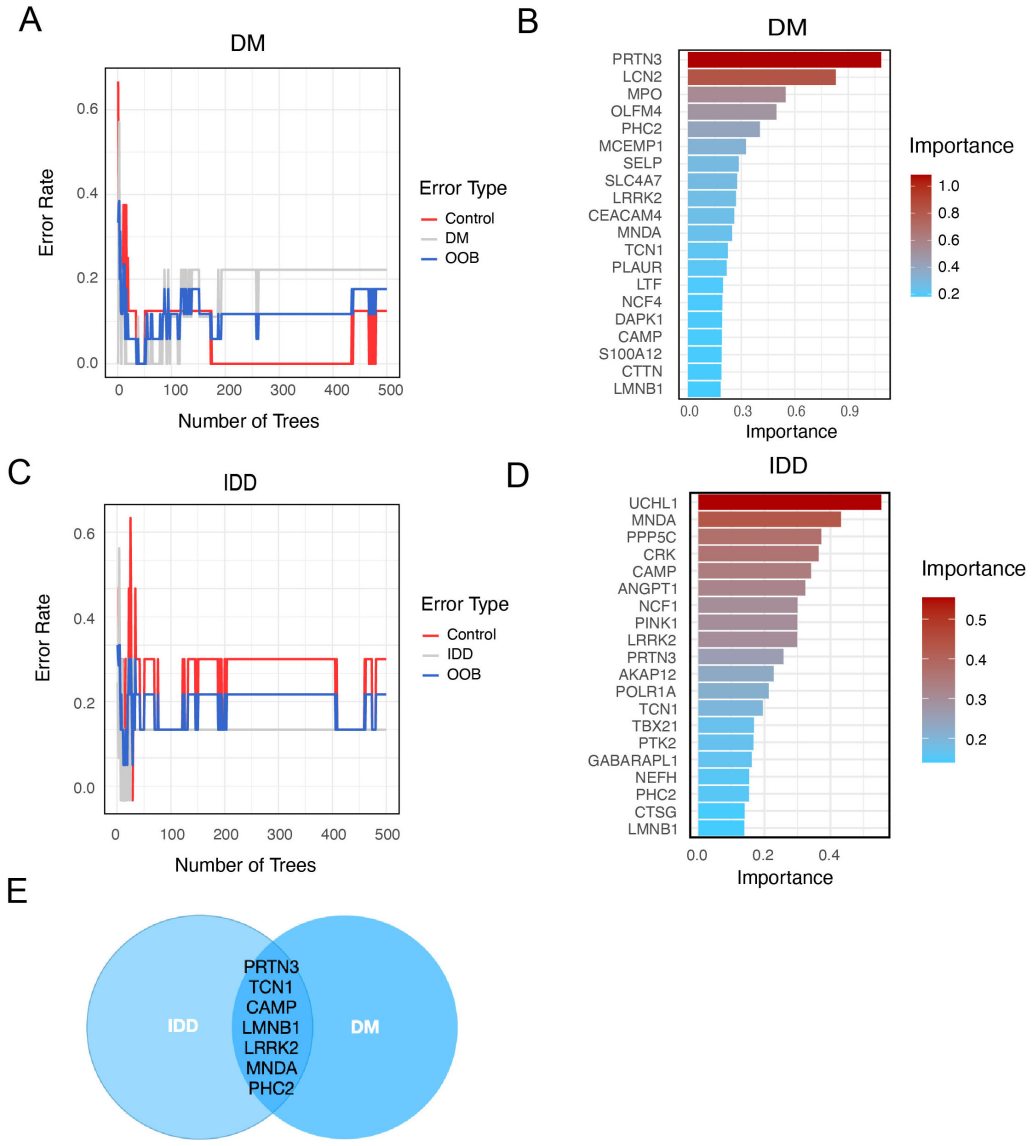
Identify potential shared diagnostic genes based on machine learning. **(A)**. Curve of Random Forest (RF) error rate in the diabetes (DM) group. The line plot displays the error rate of the RF model applied to the DM group. The y-axis represents the error rate, while the x-axis indicates the number of decision trees used in the model. **(B)**. Top 20 most important genes identified by RF in the DM group. **(C)**. Line plot of the RF error rate in the intervertebral disc degeneration (IDD) group. The line plot displays the error rate of the RF in the IDD group. The y-axis represents the error rate, while the x-axis indicates the number of decision trees used in the model. **(D)**. Barplot of top 20 genes ranked by importance in IDD group. **(E)**. Venn diagram showing overlapping RF-identified genes in both IDD and DM.

For further selection of the most promising candidate diagnostic gene targets with significant potential for classifying the disease and control groups, we applied Random Forest (RF) algorithms based on the 47 core interaction proteins. In the IDD group, the 47 core proteins were input into the RF classifier, and the top 20 genes were ranked according to their importance (**Figures 4A, B**). Similarly, in the diabetes mellitus (DM) group, the RF machine learning screening process identified the top 20 potential biomarkers (**Figures 4C, D**). By overlapping the top 20 most important genes from both the IDD and DM groups, we identified PRTN3, TCN1, CAMP, LMNB1, LRRK2, MNDA, and PHC2 as conserved, disease-shared genes with potential as hub diagnostic biomarkers (**Figure 4E**).

### 3.4 Diagnostic value and validation of diagnostic hub biomarkers

**Figure 5:**
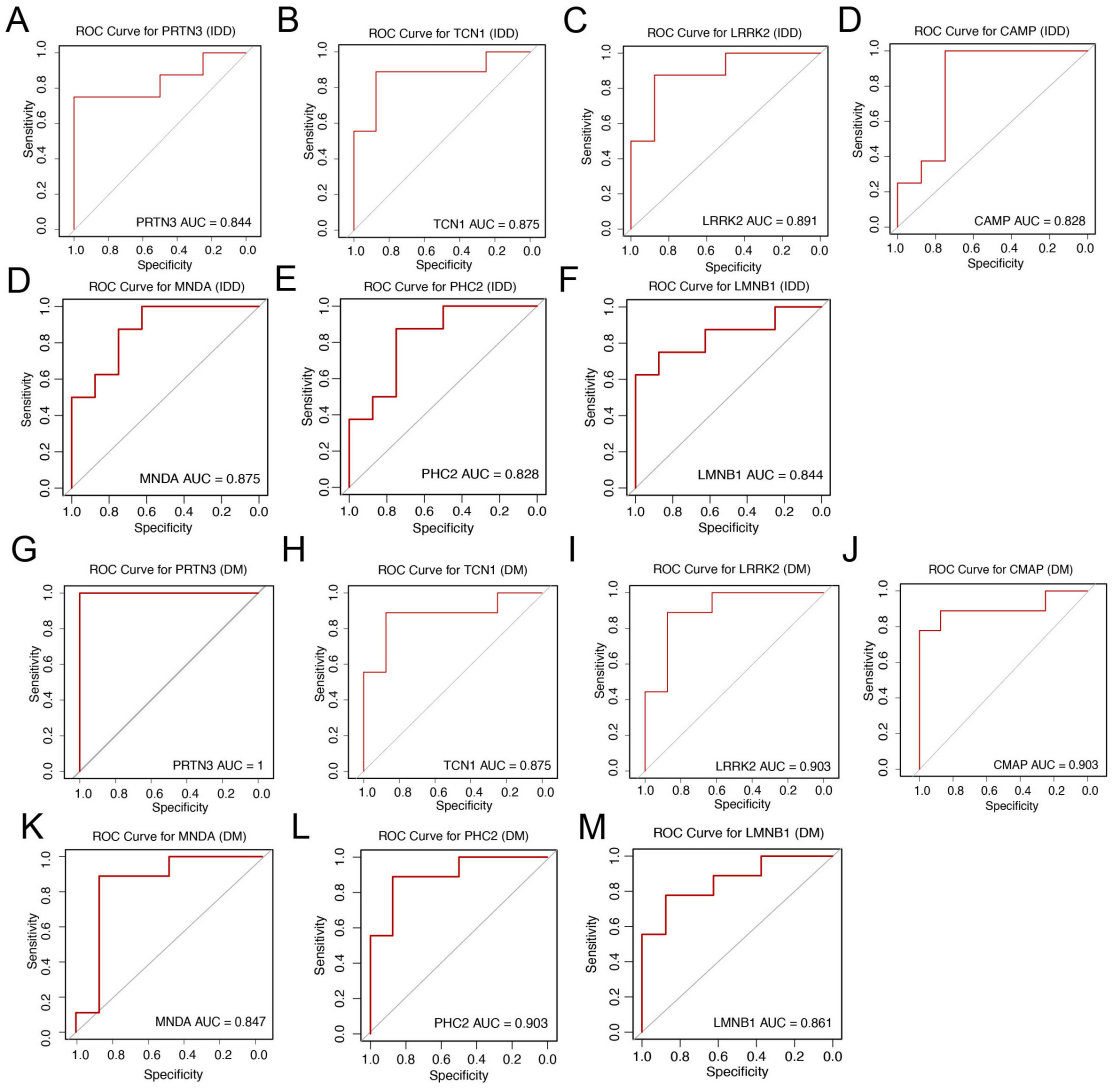
Diagnostic value and validation of diagnostic hub biomarkers. **(A–F)** Evaluation of the diagnostic performance of hub biomarkers using Area Under the Curve (AUC) analysis. The AUC values derived from Receiver Operating Characteristic (ROC) curves for PRTN3, TCN1, LRRK2, CAMP, MNDA, PHC2, and LMNB1 are shown, respectively, to assess their discriminatory power between IDD patients and controls. **(G–M)** Assessment of the diagnostic efficacy of hub biomarkers for DM. The AUC values derived from Receiver Operating Characteristic (ROC) curves for PRTN3, TCN1, LRRK2, CAMP, MNDA, PHC2, and LMNB1 are shown, respectively, to evaluate their diagnostic capacity in distinguishing DM patients from controls.

To assess the diagnostic capability of the seven core genes for intervertebral disc degeneration (IDD) and diabetes mellitus (DM), Receiver Operating Characteristic (ROC) analysis was performed. The area under the ROC curve (AUC) values, which reflect the discriminatory power of each gene, were as follows: For IDD: PRTN3 (0.844), TCN1 (0.875), LRRK2 (0.891), CAMP (0.828), MNDA (0.875), PHC2 (0.828), and LMNB1 (0.844) (**Figures 5A–F**). For DM: PRTN3 (1.000), TCN1 (0.875), LRRK2 (0.903), CAMP (0.903), PHC2 (0.903), MNDA (0.847), and LMNB1 (0.861) (**Figures 5G–M**). These results suggest that all seven genes demonstrate strong diagnostic potential for both IDD and DM, effectively distinguishing affected individuals from healthy controls.

### 3.5 Expression validation of core diagnostic genes

**Figure 6:**
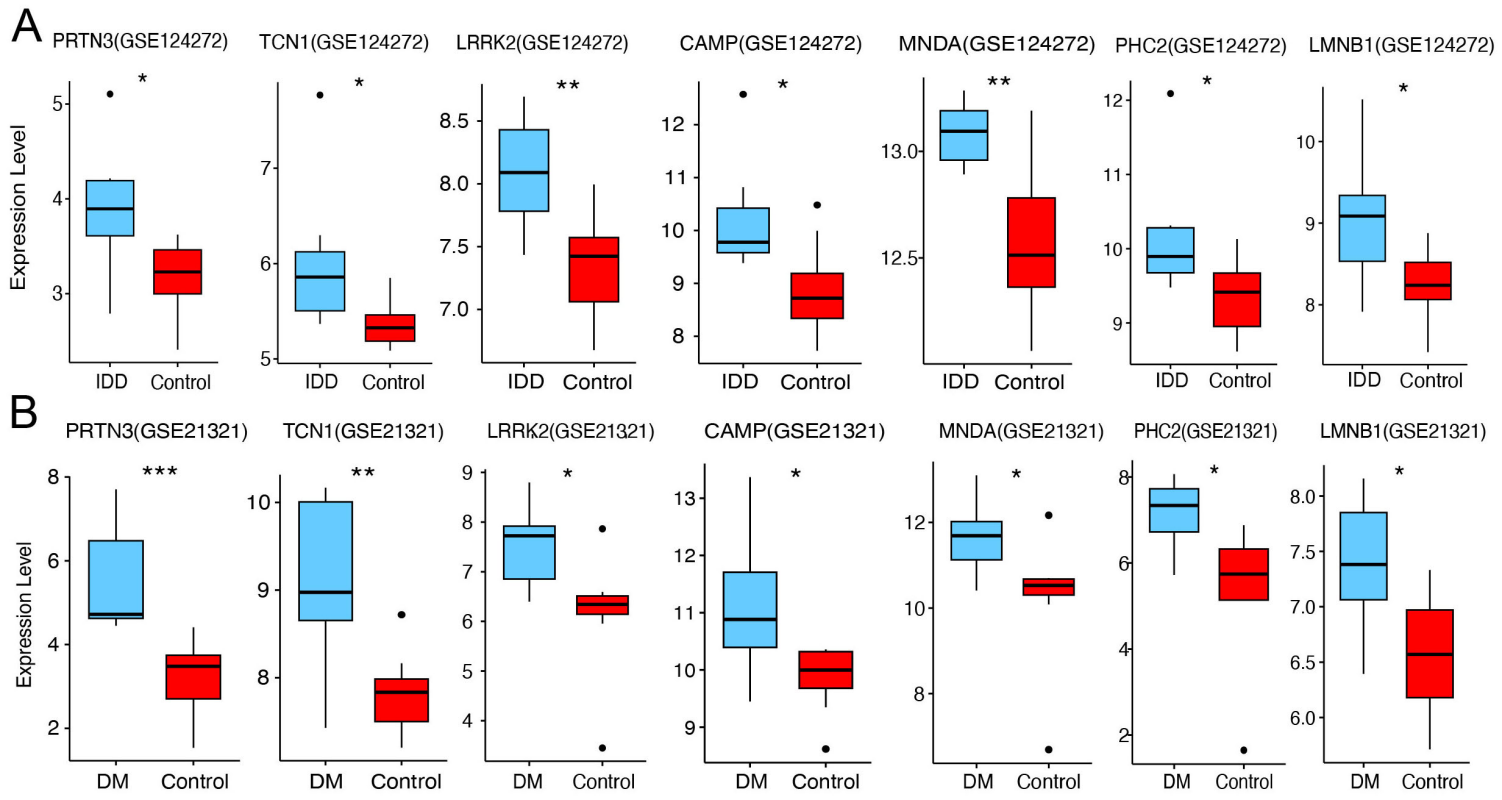
Expression validation of core diagnostic genes. **(A)** Boxplot of core diagnostic gene expression in the IDD group and healthy control. **(B)** Boxplot of core diagnostic gene expression in the DM group and healthy control. Statistical significance was assessed using a t-test (*P < 0.05, **P < 0.01, ***P < 0.001).

To further validate the expression of the seven core diagnostic genes (PRTN3, TCN1, LRRK2, CAMP, MNDA, PHC2, and LMNB1), we analyzed the corresponding discovery datasets for IDD (GSE123272) and DM (GSE21321). Compared to normal samples, all seven genes were significantly upregulated in both the IDD (**Figure 6A**) and DM (**Figure 6B**) cohorts (p < 0.05), which is consistent with our previous findings.

### 3.6 Experimental validation of PRTN3 expression in clinical samples

**Figure 7:**
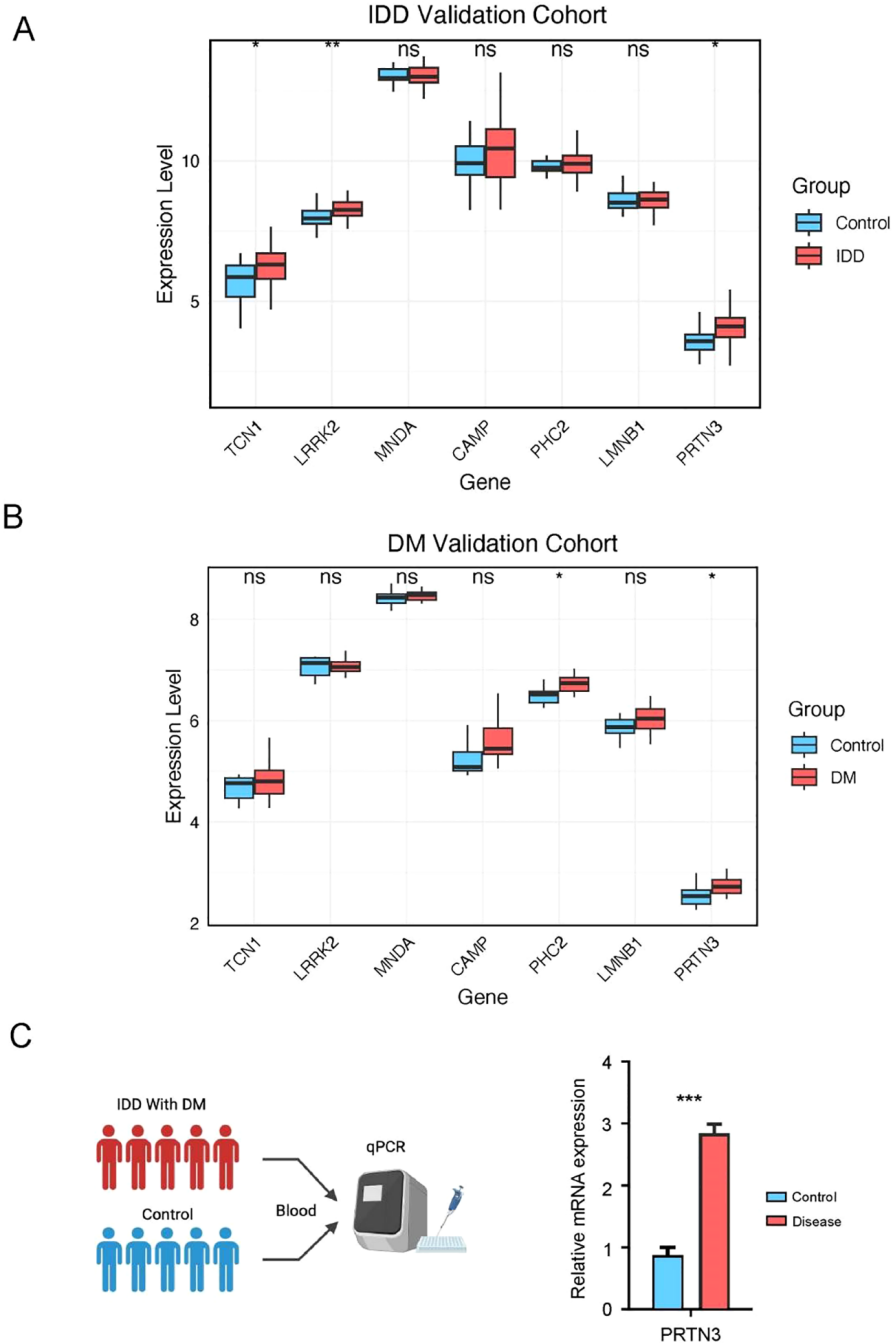
Experimental validation of PRTN3 expression in clinical samples. **(A)** Boxplot of core diagnostic gene expression in the IDD group and healthy control. **(B)** Boxplot of core diagnostic gene expression in the DM group and healthy control. **(C)** This panel illustrates the relative mRNA expression levels of PRTN3 in patients with concurrent intervertebral disc degeneration (IDD) and diabetes mellitus (DM) compared to healthy controls. The y-axis represents the relative expression level of PRTN3, while the x-axis differentiates between the IDD with DM group and the control group. Statistical significance was assessed using a t-test (*P < 0.05, **P < 0.01, ***P < 0.001).

To further assess the diagnostic potential of these genes, we utilized an external IDD validation cohort. The results demonstrated significant upregulation of TCN1, LRRK2, and PRTN3 in the IDD group (p < 0.05), while the other core diagnostic genes exhibited an upward trend that did not reach statistical significance (**Figure 7A**). In the DM validation cohort, the expression of PHC2 and PRTN3 was significantly elevated in the disease group (**Figure 7B**). Notably, PRTN3 exhibited a consistent upregulation pattern across both the discovery and validation cohorts for both IDD and DM, suggesting its potential as a key biomarker with significant biological relevance in both diseases. As anticipated, in our internal cohort of patients with concurrent IDD and DM, PRTN3 expression in blood samples was also significantly upregulated (**Figure 7C**). In summary, these findings strongly support the hypothesis that PRTN3 serves as a conserved biomarker for both IDD and DM, highlighting its diagnostic and biological importance.

### 3.7 Pathway enrichment associated with PRTN3

**Figure 8:**
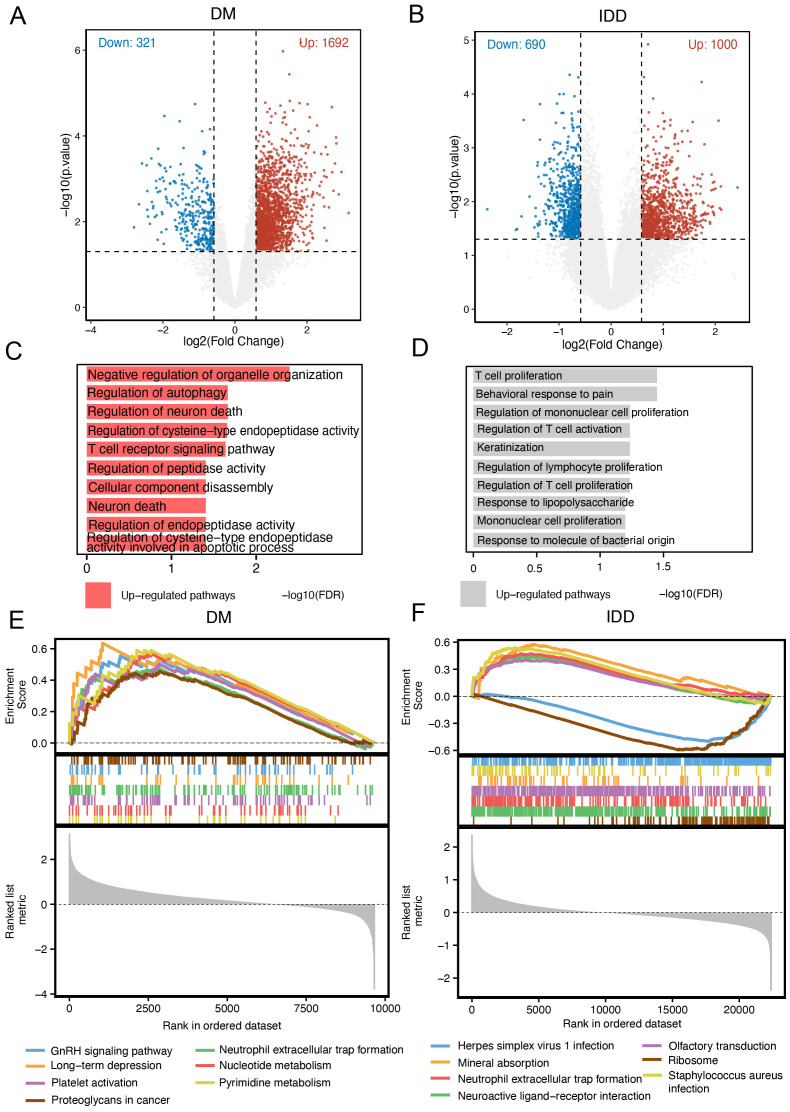
Pathway enrichment associated with PRTN3. **(A)** Volcano plot of differentially expressed genes (DEGs) in the DM cohort: Red dots represent significantly upregulated genes in patients with high PRTN3 expression, blue dots represent significantly downregulated genes, and gray dots represent genes without significant changes. **(B)** Volcano plot of DEGs in the IDD cohort: Red dots indicate significantly upregulated genes, while blue dots indicate significantly downregulated genes in patients with high PRTN3 expression. **(C)** GO_BP pathways enriched in the DM cohort for upregulated genes. **(D)** GO_BP pathways enriched in the IDD cohort for upregulated genes. **(E)** GSEA analysis for the DM cohort stratified by PRTN3 expression: Pathways with an enrichment score greater than 0 indicate activation of KEGG pathways in patients with high PRTN3 expression. **(F)** GSEA analysis for the IDD cohort stratified by PRTN3 expression: Pathways with an enrichment score greater than 0 represent activation of KEGG pathways in the high PRTN3 expression group.

Given the consistent expression trends of PRTN3 observed across multiple cohorts, we further investigated its role in intervertebral disc degeneration (IDD) and diabetes mellitus (DM). Patients in the IDD and DM groups were stratified into high- and low-PRTN3-expression subgroups based on the average expression levels. Differentially expressed genes (DEGs) were identified using the limma R package. In the DM group, 321 genes were downregulated and 1,692 genes were upregulated (**Figure 8A**, **Supplementary Table 1**), whereas in the IDD group, 690 genes were downregulated and 1,000 genes were upregulated (**Figure 8B**, **Supplementary Table 1**). To explore the associated biological functions, we performed Gene Ontology Biological Process (GO-BP) enrichment analysis on the upregulated and downregulated gene sets. The results indicated that only the upregulated genes exhibited significant pathway enrichment. In the DM group, the top enriched pathways included negative regulation of organelle organization, regulation of autophagy, regulation of neuron death, regulation of cysteine-type endopeptidase activity, and the T cell receptor signaling pathway (**Figure 8C**). In the IDD group, multiple immune-related pathways such as T cell proliferation, regulation of T cell activation, regulation of lymphocyte proliferation, and regulation of T cell proliferation were significantly enriched (**Figure 8D**). These findings are consistent with previous analyses and align with the pathophysiological features of the diseases, highlighting the pivotal role of PRTN3 in modulating immune-related pathways that may contribute to the shared mechanisms of IDD and DM. Furthermore, Gene Set Enrichment Analysis (GSEA) was performed to evaluate KEGG pathway enrichment in relation to PRTN3 expression. In the DM group, neutrophil-related pathways were significantly enriched, while in the IDD group, neutrophil extracellular trap (NET) formation was notably enriched (**Figures 8E, F**). These results suggest that immune-related pathways, particularly those involving neutrophils, may serve as crucial mediators in the PRTN3-regulated shared mechanisms of IDD and DM. This underscores the critical role of PRTN3 in the shared pathogenesis of these conditions.

### 3.8 Immune infiltration analysis reveals the association between PRTN3 and neutrophils

**Figure 9:**
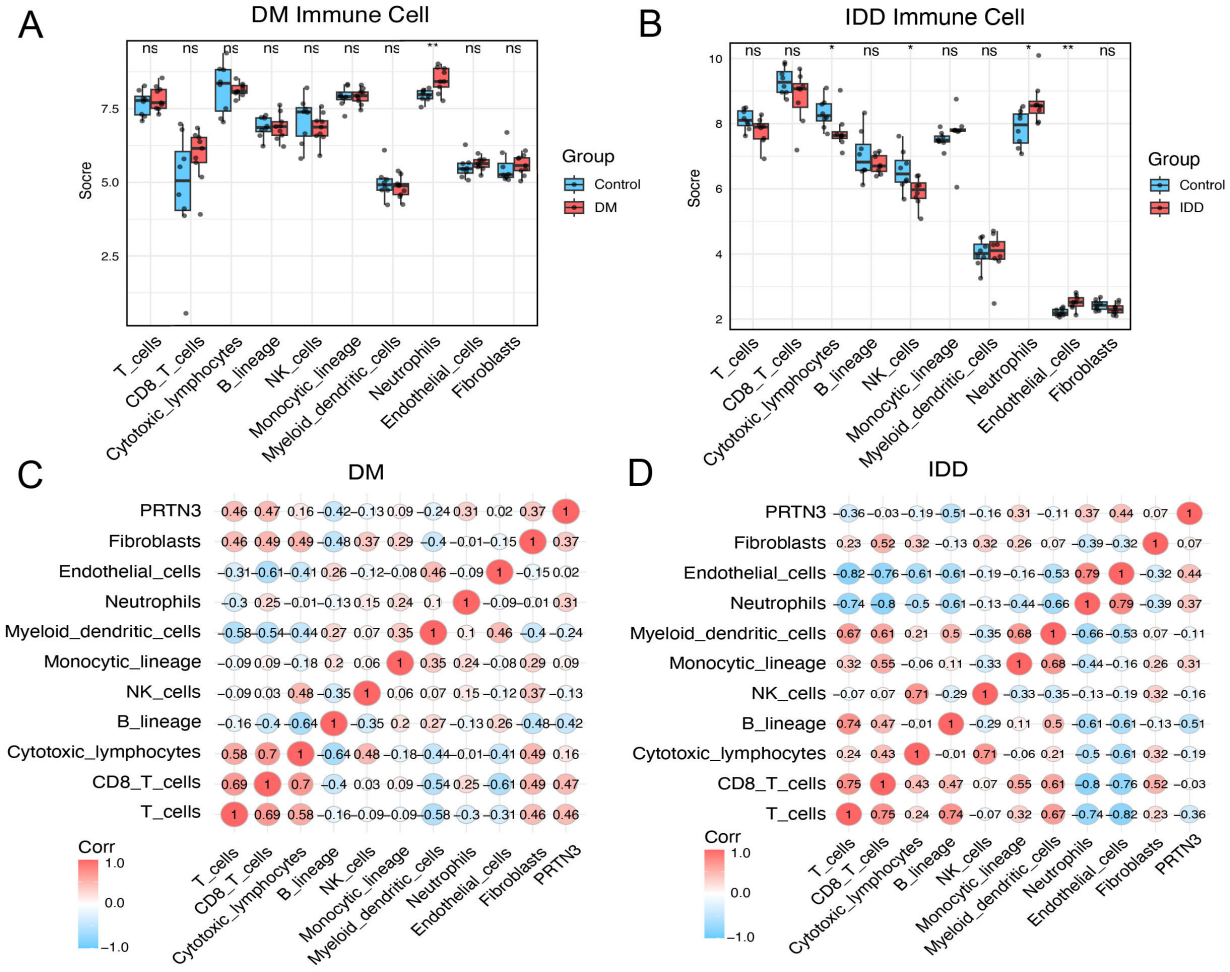
Immune infiltration analysis reveals the association between PRTN3 and neutrophils. **(A)** Boxplot of immune cell score in DM cohort. **(B)** Boxplot of immune cell score in IDD cohort. **(C)** Pearson correlation heatmap across PRTN3 and immune cells in the DM cohort. **(D)** Pearson correlation heatmap across PRTN3 and immune cells in the IDD cohort. (*P < 0.05, **P < 0.01).

Given the significant association between PRTN3 expression and immune-related pathways, particularly neutrophil-associated pathways, the interplay between PRTN3 and immune cell infiltration was further investigated. The MPCounter algorithm was applied to estimate immune cell enrichment in both the diabetes mellitus (DM) and intervertebral disc degeneration (IDD) cohorts. In the DM cohort, neutrophils were significantly enriched in patients compared to healthy controls (P < 0.01) (**Figure 9A**). Similarly, in the IDD cohort, patients also exhibited significant neutrophil enrichment (P<0.05). Notably, B cells and endothelial cell types were also significantly upregulated in the IDD group (**Figure 9B**). Remarkably, the consistent upregulation of neutrophils in both DM and IDD highlights their potential role in disease progression. The association between PRTN3 and immune cells was further quantified. As anticipated, the Pearson correlation coefficients between PRTN3 and neutrophils were “0.31” in the DM cohort and “0.37” in the IDD cohort (**Figures 9C, D**). These findings suggest that PRTN3 may contribute to the shared pathogenesis of DM and IDD through the regulation of neutrophil activity.

## 4 Discussion

The clinical association between intervertebral disc degeneration (IDD) and diabetes mellitus (DM) has been confirmed by multiple clinical studies (8, 18, 19). However, despite this established association, the molecular crosstalk underlying the mechanistic connection between IDD and DM remains unexplored. In this study, the comorbid mechanisms and shared biomarkers of IDD and DM were investigated.

By integrating transcriptomic data from IDD and DM, differentially expressed genes (DEGs) associated with both diseases were systematically identified. In-depth protein-protein interaction (PPI) analysis highlighted the critical role of immune-related pathways in the crosstalk of IDD and DM. Through the integration of core interacting proteins and a Random Forest algorithm, seven potential biomarkers for IDD and DM were identified, including PRTN3, TCN1, LRRK2, MNDA, CAMP, PHC2, and LMNB1. Importantly, PRTN3 exhibited a consistent expression trend across both the available discovery and validation cohorts. Furthermore, in internally collected blood samples from patients with both IDD and DM, PRTN3 was significantly upregulated compared to healthy controls. This finding supports the potential of PRTN3 as a diagnostic biomarker for both DM and IDD.

Notably, our immune cell analysis revealed increased neutrophil infiltration in both IDD and DM, highlighting the potential role of neutrophils in the pathophysiological interplay between these conditions. Consistent with our findings, neutrophil activation is a hallmark of many inflammatory diseases. Moreover, in type 2 diabetes mellitus (T2DM), neutrophil levels are chronically elevated (20). Neutrophil extracellular traps (NETs) play a critical role in the development of various diabetic complications. In T2DM, NET release products are elevated, accompanied by increased double-stranded DNA (dsDNA) levels (21), which are strongly associated with cardiovascular diseases and diabetic nephropathy. In the context of intervertebral disc degeneration (IDD), neutrophil infiltration into the extracellular matrix contributes to disease progression through the MIF/ACKR3 axis. This underscores the role of neutrophils in mediating the molecular crosstalk between diabetes and IDD (22, 23). Notably, our study provides strong evidence of a consistent positive correlation between PRTN3 expression and neutrophil abundance in both IDD and DM.

Proteinase 3 (PRTN3) is a neutrophil serine protease. Ample evidence supports the critical role of PRTN3 in neutrophil-associated inflammation (24). Studies have shown that PRTN3 deletion reduces IC-mediated neutrophil infiltration and activation *in vitro*. Furthermore, PRTN3 enhances neutrophil-dependent inflammation by eliminating the anti-inflammatory activity of progranulin (PGRN) (25). It also plays a role in neutrophil transendothelial migration by interacting with CD177 and CD31, influencing the efficiency of extravasation (26). This interaction suggests a regulatory role of PR3 in the efficiency of neutrophil migration. Additionally, PRTN3 exerts a pro-inflammatory effect through its interaction with the complement pathway. Specifically, PRTN3 cleaves the complement component 5a receptor (C5aR) on the neutrophil surface, leading to the loss of its N-terminal region and preventing its binding to C5a, thereby modulating neutrophil-mediated inflammation (27). Therefore, our data provide valuable evidence supporting the role of PRTN3 in regulating neutrophil-associated inflammation, which may help to mediate the crosstalk between DM and IDD.

Overall, our study identified shared gene signatures between IDD and DM using an integrated bioinformatics approach. By combining protein interaction analysis with machine learning, we identified seven potential biomarkers and validated the consistent upregulation of PRTN3 across multiple datasets and internal samples. Further analyses indicated that PRTN3 may be involved in neutrophil regulation, potentially contributing to the comorbidity mechanism of IDD and DM. Therefore, our study provides valuable insights into the potential comorbidity mechanisms and diagnostic possibilities for patients with concurrent IDD and DM.

## 5 Conclusion

In this study, we utilized bioinformatics approaches to elucidate the molecular crosstalk between intervertebral disc degeneration (IDD) and diabetes mellitus (DM). Our findings highlight shared immune-related pathways, particularly involving neutrophil activity—that contribute to the pathogenesis of both diseases. Additionally, we identified PRTN3 as a conserved diagnostic gene for IDD and DM and established its association with neutrophil activity. These results suggest that PRTN3 may serve not only as a promising diagnostic biomarker but also holds potential as a therapeutic target for comorbid IDD and DM.

## Data Availability

The original contributions presented in the study are included in the article/**Supplementary Material**. Further inquiries can be directed to the corresponding author.
